# Assessing the Efficacy of Three Hatchery Disinfectants for the Inactivation of a Lake Sturgeon Herpesvirus (Family: *Alloherpesviridae*)

**DOI:** 10.3390/v16071062

**Published:** 2024-06-30

**Authors:** Amber E. Johnston, Megan A. Shavalier, Kim T. Scribner, Esteban Soto, Susan Yun, Thomas P. Loch

**Affiliations:** 1 Aquatic Animal Health Laboratory, Aquatic Animal Disease Ecology Program, Michigan State University, East Lansing, MI 48824, USA; 2 Department of Fisheries and Wildlife, College of Agriculture and Natural Resources, Michigan State University, East Lansing, MI 48824, USA; 3 Department of Epidemiology, School of Veterinary Medicine, University of California-Davis, Davis, CA 95616, USA; 4 Department of Pathobiology and Diagnostic Investigation, College of Veterinary Medicine, Michigan State University, East Lansing, MI 48824, USA

**Keywords:** lake sturgeon, herpesvirus, disinfectant, biosecurity, Great Lakes

## Abstract

Infectious diseases are a leading cause of losses in the aquaculture industry and conservation programs globally. Simultaneously, infectious diseases pose a substantial risk to fish being hatchery-reared and released into natural habitats for conservation purposes, including the Great Lakes lake sturgeon (*Acipenser fulvescens*, i.e., GL-LST). Recently, an alloherpesvirus (lake sturgeon herpesvirus 2, i.e., LSHV-2) capable of inducing disease and/or mortality in adult and juvenile GL-LSTs was detected in two adult GL-LST populations. To begin developing disease prevention and/or control methods, in vitro experiments were designed to determine the susceptibility of LSHV-2 to disinfectants commonly used in hatchery and aquaculture facilities (Virkon^®^-Aquatic: potassium peroxymonosulfate; Ovadine^®^: polyvinylpyrrolidone iodine complex; and Perox-Aid^®^: hydrogen peroxide). Cultured LSHV-2 was exposed to each disinfectant at two concentrations (Virkon^®^-Aquatic: 0.5% and 1%; Ovadine^®^: 50 and 100 ppm; and Perox-Aid^®^: 500 and 1000 ppm) in duplicate for durations of 1, 10, and 30 min. Following exposure, the disinfectant was neutralized, and after a 14-day incubation period on a white sturgeon × lake sturgeon hybrid cell line (WSxLS), percent reduction was calculated by comparing the 50% tissue culture infectious doses (TCID_50_/mL) of the virus with and without disinfectant exposure. When exposed to Perox-Aid^®^, LSHV-2 percent reduction ranged from 58.7% to 99.5%. When exposed to Ovadine^®^, the percent reduction ranged from 99.4% to 100%. Lastly, the percent reduction when exposed to Virkon^®^-Aquatic was 100% for both concentrations and all timepoints. The results herein provide evidence that both Virkon^®^-Aquatic and Ovadine^®^ are virucidal to LSHV-2 and may represent a means to reduce virus transmission risk under field settings.

## 1. Introduction

Infectious disease outbreaks can lead to sizeable losses in facilities rearing fish for conservation, stock enhancement, and/or consumption (e.g., hatcheries and aquaculture facilities). The 2018 Census of Aquaculture listed disease as the leading cause of production losses in aquaculture facilities [1]. A cornerstone for preventing pathogen transmission within and between aquaculture facilities and hatcheries is biosecurity, generally defined as preventative measures aimed at reducing risks associated with infectious agents in a facility, including their transmission within and between facilities and/or dissemination from or into the environment [2]. 

Among the groups of microbial fish pathogens that have been linked to heavy losses in captive-reared fishes are alloherpesviruses (family: *Alloherpesviridae*), which cause significant disease and mortality in a range of fishes [3], including sturgeons (family: *Acipenseridae*) [4,5,6]. Within the *Alloherpesviridae* family, two recently characterized viruses have been detected in wild lake sturgeon (e.g., lake sturgeon herpesviruses, i.e., LSHVs) [6,7], with at least one (e.g., lake sturgeon herpesvirus-2) shown to be capable of causing disease and mortality (~33% cumulative mortality) in experimentally exposed juvenile lake sturgeons [6]. 

For the LSHVs and most other alloherpesviruses affecting fish, prevention and control options are limited, making avoidance and reductions in virus transmission risk primary pillars of fish health management plans. Further, other alloherpesviruses have been recovered from reproductive fluids/tissues [4] and are suspected, or proven, to be transmitted vertically [8]. Among the alloherpesviruses isolated from reproductive fluids and/or suspected of vertical transmission in sturgeon is Acipenserid Herpesvirus 2, which was detected in the ovarian fluid of spawning-age white sturgeon, and is a close relative to LSHVs [4,6,7]. In this context, the disinfection of both fomites (e.g., gear used under field settings, nets, hoses, etc.) and gametes collected from wild fishes is often undertaken to reduce the risk of viruses gaining access to captive-reared fishes and/or spreading within aquaculture facilities and hatcheries [9].

Multiple disinfectants are used for the disinfection of aquaculture and hatchery facilities in the USA, including Virkon^®^-Aquatic (Syndel, Ferndale, WA, USA), which is a potassium peroxymonosulfate-based disinfectant that has wide-ranging bactericidal and virucidal activity [10], is relatively safe for fish [11], and has recently been shown to be promising in reducing risk by the fomite transmission of salmonid herpesvirus 3 (*Alloherpesviridae*) [12]. Similarly, the polyvinylpyrrolidone–iodine (PVP iodine) complex (e.g., Ovadine^®^; Syndel, Ferndale, WA, USA) is widely utilized to disinfect fertilized fish eggs in an effort to prevent pathogen transmission from broodstock to progeny via infected reproductive fluids [9,13], including in facilities raising lake sturgeon that are often populated using gametes from wild broodstock [14]. Additionally, hydrogen peroxide (e.g., Perox-Aid^®^; Syndel, Ferndale, WA, USA) is routinely used to treat multiple external infections in various fish life stages (eggs, fry, fingerlings, etc.) via immersion/bath treatments, including in lake sturgeon [15]. Unfortunately, given the recent discovery of the LSHVs [6,7], it remains unknown if these three commonly used hatchery disinfectants are capable of inactivating these viruses at the currently recommended concentrations and durations. 

With the long-term goal of equipping personnel studying and/or raising lake sturgeon with efficacious means of reducing lake sturgeon herpesvirus transmission risk, this study aimed to assess the efficacy of Virkon^®^-Aquatic, Ovadine^®^, and Perox-Aid^®^ in vitro using a recently recovered and well-characterized isolate of LSHV-2 [6].

## 2. Materials and Methods

### 2.1. Cell Culture

To support virus detection and propagation, a white sturgeon x lake sturgeon spleen (WSxLS) cell line was used according to previously published protocols [6,16]. Growth media for WSxLS cells (MEM-10-SBH) was comprised of Eagle’s minimum essential media (with Earle’s salts, nonessential amino acids, and sodium pyruvate (ATCC, Manassas, VA, USA)) supplemented with 10% tryptose phosphate broth (ThermoFisher Scientific, Waltham, MA, USA), 10% fetal bovine serum (Gemini BioProducts, Sacramento, CA, USA), penicillin (100 IU mL^−1^), streptomycin (100 µg mL^−1^), amphotericin B (2.5 µg mL^−1^), and L-Glutamine (2 mM) and buffered with sodium bicarbonate and HEPES (Fisher Scientific, Waltham, MA, USA) to a pH of 7.4–7.6. For virus propagation, cells were grown at 21 °C in 96-well flat-bottom plates (Corning, Corning, NY, USA) to 80–90% confluency (<48 h) in MEM-2-TH, a medium identical to MEM-10-SBH except for a modification to contain 2% rather than 10% FBS and buffered with HEPES and UltraPure Tris (Invitrogen, Carlsbad, CA, USA) to a pH of 7.4–7.6 [17].

### 2.2. Virus Preparation

Lake sturgeon herpesvirus-2 isolate 200413-11TC was originally isolated from a skin lesion of an adult Great Lakes lake sturgeon collected from Black River on WSxLS cells [6]. The isolate was passaged five times on WSxLS cells prior to freezing in liquid nitrogen in a solution of 20% FBS and 20% glycerol [6]. All disinfectant efficacy experiments utilized this isolate following the methods outlined below. The virus isolate was rapidly thawed from cryopreservation (−193 °C) at 25 °C. The virus stock was then gently mixed and inoculated into multiple wells of a 96-well plate (Corning, Corning, NY, USA) with an 80–90% confluent monolayer of WSxLS cells and incubated at 15 °C. Following the development of cytopathic effects (CPE), cells and the supernatant were harvested and passaged in a 25 cm^2^ cell culture flask (Corning, Corning, NY, USA) containing an 80–90% confluent monolayer of WSxLS cells, incubated again at 15 °C, and monitored for the development of CPE. Eight days after inoculation in the 25 cm^2^ flask, ~90% of cells exhibited CPE, and the cell/supernatant/virus suspension was harvested for immediate use in disinfectant experiments (Section 2.4).

### 2.3. Disinfectant Preparation

The three disinfectants (Virkon^®^-Aquatic, Ovadine^®^, and Perox-Aid^®^) were prepared at 2× the working, or final exposure, concentration for the efficacy experiments. Two solutions of each disinfectant were mixed at a high concentration and a low concentration, and all disinfectant solutions were prepared using ultrapure water. The working concentrations were determined based on the manufacturer’s guidelines for disinfectant usage. For Virkon^®^-Aquatic, solutions of 1% and 2% were prepared using the manufacturer’s instructions to obtain a working concentration of 0.5% and 1%, respectively, following the combination with the virus solution. Similarly, for Ovadine^®^, 200 ppm and 100 ppm solutions were prepared to obtain working concentrations of 100 ppm and 50 ppm, in accordance with the manufacturer’s protocols for the surface disinfection of eggs. For Perox-Aid^®^, 2000 ppm and 1000 ppm solutions were prepared (working concentrations of 1000 ppm and 500 ppm) based on the manufacturer’s recommendations. All disinfectant solutions were filter-sterilized through a 0.22 µM filter (Santa Cruz Biotechnology, Dallas, TX, USA) prior to use and prepared immediately prior to the start of the experiments. Prior to all efficacy experiments and to visually check for any cytotoxicity directly induced by the disinfectants on the WSxLS cells, all disinfectant working concentrations were inoculated onto WSxLS cells and examined for cytotoxicity via light microscopy routinely for 14 days. 

### 2.4. Virus Exposure to the Disinfectants

For each disinfectant concentration, two aliquots of 500 µL of virus cell/supernatant suspension were prepared as duplicates (Section 2.2). Next, 500 µL of 2× disinfectant stock (Section 2.3) was aliquoted into each virus aliquot tube to achieve the desired working concentration. Immediately after the addition of the disinfectant to the virus suspension and gentle mixing, a timer was started and virus/disinfectant aliquots were incubated at ambient temperature (~22 °C). Three disinfectant exposure durations were assessed per disinfectant concentration and duplicate: 1, 10, and 30 min. At each respective time point, the disinfectant was deactivated following protocols detailed by Amend and Pietch [18]. In brief, 50 µL of virus/disinfectant suspension was aliquoted into 450 µL of MEM-10-SBH and gently mixed. Next, 100 µL of the 1:10 diluted virus/disinfectant suspension was aliquoted into 400 µL of MEM-10-SBH for a final dilution of 1:50. Control aliquots of the virus not exposed to the disinfectant were also diluted to a ratio of 1:1 with MEM-2-TH to simulate the initial dilution of the disinfectant and then diluted to a ratio of 1:50 with MEM-10-SBH or 1:50 with MEM-2-SBH. The dilution with MEM-2-SBH was to ensure that dilution with MEM-10-SBH did not affect virus growth due to a 10% FBS concentration. 

Following the deactivation of disinfectants and to estimate the active virus concentration, a 50% tissue culture infectious dose (TCID_50_/mL) was calculated. Seven 10-fold serial dilutions were prepared, using MEM-2-TH, from each deactivated disinfectant/virus suspension and the controls, inoculated onto 96-well plates (four wells per dilution), and subsequently monitored for the development of CPEs via light microscopy. Likewise, each day that the experiments were performed, working concentrations of each disinfectant (50 and 100 ppm for Ovadine^®^, 500 and 1000 ppm for Perox-Aid^®^, and 0.5% and 1% Virkon^®^-Aquatic) were inoculated in quadruplicate onto WSxLS cells and monitored for 14 days to screen for visual indications of disinfectant-induced cytotoxicity. All TCID_50_/mL plates were incubated at 15 °C and checked periodically for 14 days. After 14 days, the TCID_50_/mL plates were examined for CPEs, and wells exhibiting CPEs consistent with that of LSHV-2 [6] were deemed positive for active virus. All TCID_50_/mL values were calculated according to Reed and Muench [19]. Virus concentrations (TCID_50_/mL) for each disinfectant concentration at each time point (Table 1) were calculated by averaging the TCID_50_/mL values for each replicate.

### 2.5. Calculation of the Percent Reduction in Active Virus

To calculate the percent reduction in active virus for each disinfectant concentration and timepoint, mean TCID_50_/mL values of experimental disinfectant exposure groups and mean non-disinfectant-exposed virus TCID_50_/mL values were calculated. Next, the experimental exposure group TCID_50_/mL mean was divided by the respective non-disinfectant-exposed TCID_50_/mL mean and multiplied by 100 to calculate the percent reduction.

## 3. Results

### 3.1. Initial Virus Concentration and Disinfectant Control Results

The concentration of LSHV-2 isolate 200413-11TC for these experiments, as determined by TCID_50_/mL analyses, was 2.39 × 10^5^ (Table 1) and did not differ when diluted with MEM-10-SBH or MEM-2-TH. Throughout the experiments, no cytotoxic effects were noted on cells inoculated with the 1× concentration of any disinfectant.

### 3.2. Efficacy of Disinfectants against LSHV-2

When LSHV-2 isolate 200413-11TC was exposed to Virkon^®^-Aquatic (0.5% and 1.0%), no cytopathic effects were observed at any of the three timepoints, indicative of complete (100%) active virus percent reduction (Table 1 and Table 2). Following exposure to Ovadine^®^ at a concentration of 50 ppm, a 99.35% reduction in active LSHV-2 occurred after just one minute (Table 1 and Table 2), with later exposure time points (i.e., 10 and 30 min) yielding 99.9% and 100% reductions, respectively (Table 1 and Table 2). Similarly, when exposed to a final concentration of 100 ppm Ovadine^®^, >99% reduction was noted after all three exposure times (Table 1 and Table 2). Lastly, when LSHV-2 isolate 200413-11TC was exposed to Perox-Aid^®^ at a concentration of 500 ppm, a 58.7% reduction in active virus was observed after 1 min of exposure (Table 1 and Table 2). After 10 and 30 min of exposure, decreases of 92.3% and 97.1% were observed (Table 1 and Table 2), respectively. At 1000 ppm, a 92.7% reduction in active virus was observed after just one minute of exposure, while 96.0% and 99.5% reductions were observed after 10 and 30 min of exposure, respectively (Table 1 and Table 2).

## 4. Discussion

Prior to this study and given the relatively recent discovery of the lake sturgeon herpesviruses [6,7], disinfectant efficacy data for LSHVs were lacking, leaving personnel raising and/or studying lake sturgeon in the field with little information to guide potential disinfection options aimed at reducing the risk of virus spread. Herein, albeit to varying degrees, laboratory-based experiments provide evidence that potassium peroxymonosulfate-based, polyvinylpyrrolidone–iodine complex-based, and hydrogen peroxide-based disinfectants are capable of reducing lake sturgeon herpesvirus (i.e., LSHV-2) titers under in vitro conditions. Although the present study did not evaluate the inactivation efficacy of these three compounds under field conditions, the results show that at least Virkon^®^-Aquatic and Ovadine^®^ represent potentially promising tools for reducing LSHV transmission risk.

Among the three assayed disinfectants, Virkon^®^-Aquatic most rapidly and effectively led to a depletion of detectable replicating LSHV-2. Indeed, after just one minute of exposure to Virkon^®^-Aquatic at both 0.5% and 1%, no evidence of actively replicating virus could be detected, thus showing true virucidal activity against enveloped viruses according to international standards (i.e., ≥four-log [≥99.99%] reduction) [20]. Although Virkon^®^-Aquatic is not used to treat fish or eggs directly, it is widely used in hatchery and field settings for the disinfection of fomites [12]. Further, it is safe for use in environments housing fish (e.g., tanks or raceways) [11]. The virucidal effects of Virkon^®^-Aquatic and other potassium peroxymonosulfate-based disinfectants are widely studied, and this disinfectant is efficacious in reducing transmission risk for a diversity of viruses [21,22,23], including another alloherpesvirus, namely, salmonid herpesvirus 3 [12].

Surface disinfection of fish eggs via immersion in a PVP iodine solution is currently a widely used method for reducing the risk of pathogen transmission, including fish pathogenic viruses, via reproductive fluids and surface-contaminated eggs [9]. In this context, some alloherpesviruses are known to be transmitted through the eggs and/or reproductive fluids of infected spawning fish [8], including some that affect the sturgeon species [4]. At present, it is unknown if LSHVs are also spread via this transmission pathway, but the findings herein show that under in vitro conditions, concentrations of 50 to 100 ppm PVP iodine for 30 min led to a > five-log reduction in detectable replicating LSHV-2. Thus, it is possible that immersing lake sturgeon eggs in a similar concentration of PVP iodine for at least 30 min might lead to a decrease in lake sturgeon herpesvirus transmission risk if present in the reproductive fluids and/or on the surface of the egg. However, if transmission occurs intra-ovum, as is the case with other fish pathogens [24], egg disinfection with PVP iodine would likely be far less effective. Nevertheless, the results herein provide strong evidence that further studies exploring the effectiveness of PVP iodine-based disinfectants against LSHV transmission are warranted. Likewise, future investigations into the viral transmission and virus replicative cycles could identify if treatment at this life stage (i.e., egg), or perhaps others, would bolster disease prevention efforts.

Hydrogen peroxide (Perox-Aid^®^) is approved by the United States Food and Drug Administration Center for Veterinary Medicine to treat external infections caused by several pathogens and parasites in fish, including *Saprolegnia* spp. (causes of saprolegniasis, *Flavobacterium branchiophilum* (a cause of bacterial gill disease), and the columnaris-causing bacteria (i.e., *F. covae*, *F. columnare*, *F. davisii*, and *F. oreochromis*)) [25,26]. Indeed, hydrogen peroxide is currently used in lake sturgeon rearing facilities to control egg-associated saprolegniasis at concentrations of 500–1000 ppm for 15 min [27]. Herein, these same concentrations showed varying efficacy for reducing detectable lake sturgeon herpesvirus in vitro but, notably, did not exceed the suggested four-log reduction guideline [20]. In light of these findings and given that much remains unknown about the pathogenesis and long-term tissue targets of the lake sturgeon herpesviruses, it is uncertain if hydrogen peroxide would have any potential therapeutic effects in lake sturgeon suffering from the diseases they cause. Nevertheless, in vivo experiments by Johnston et al. [6] showed that LSHV-2 does attack and damage the skin of naïve immersion-exposed juvenile lake sturgeons. Thus, it may be worthwhile to further determine any ability of hydrogen peroxide to control LSHV infections in juvenile lake sturgeons, especially given it is the only one of the three compounds tested in this study that is approved for use in fish after hatching.

## 5. Conclusions

The results from the in vitro disinfectant efficacy experiments in this study show that Virkon^®^-Aquatic and Ovadine^®^ are capable of substantially reducing the amount of detectable LSHV-2, an alloherpesvirus recently recovered from the skin lesions of wild adult Great Lakes lake sturgeons [6]. Although the in vitro results detailed herein may not directly translate to the expected efficacy under hatchery and field settings, they highlight potential tools for reducing lake sturgeon herpesvirus transmission risk during the process of studying and raising lake sturgeon for stock enhancement and conservation purposes. To further support biosecurity efforts in lake sturgeon rearing facilities and provide critical insights into the prevention and control of LSHV-2, future experiments should aim to assess the potential efficacy of at least Virkon^®^-Aquatic and Ovadine^®^ against LSHV-2 under simulated hatchery and field conditions.

## Figures and Tables

**Table 1:** Results of 50% tissue culture infectious doses (TCID_50_/mL) for LSHV-2 isolate 200413-11TC, which was exposed to three disinfectants for three different durations. All TCID_50_/mL values are presented as the means of two experimental replicates with accompanying standard deviations. The TCID_50_/mL of LSHV-2 not exposed to disinfectant was determined to be 2.39 × 10^5^. A “0” value is indicative of no cytopathic effects being observed in either replicate.

		TCID_50_/mL		
Disinfectant	Concentration	1 min	10 min	30 min
Ovadine^®^	50 ppm	1.7 × 10^3^ ± 0.39 × 10^3^	1.4 × 10^2^ ± 0.25 × 10^2^	0
	100 ppm	1.4 × 10^3^ ± 0.32 × 10^3^	2.4 × 10^2^ ± 0.27 × 10^2^	0
Perox-Aid^®^	500 ppm	9.9 × 10^4^ ± 2.77 × 10^4^	1.8 × 10^4^ ± 0.78 × 10^4^	6.9 × 10^3^ ± 0.26 × 10^3^
	1000 ppm	1.8 × 10^4^ ± 0.49 × 10^4^	9.6 × 10^3^ ± 3.1 × 10^3^	1.1 × 10^3^ ± 0
Virkon^®^-Aquatic	0.5%	0	0	0
	1.0%	0	0	0

**Table 2:** Percent reduction in active LSHV-2 for three disinfectants, as calculated by dividing the 50% tissue culture infectious dose (TCID_50_/mL) of the virus exposed to the disinfectant by the TCID_50_/mL of the virus not exposed to each disinfectant. Percent reduction values are presented as the means of two experimental replicates.

		Percent Reduction		
Disinfectant	Concentration	1 min	10 min	30 min
Ovadine^®^	50 ppm	99.4%	99.9%	100%
	100 ppm	99.4%	99.9%	100%
Perox-Aid^®^	500 ppm	58.7%	92.3%	97.1%
	1000 ppm	93.0%	96.0%	99.5%
Virkon^®^-Aquatic	0.5%	100%	100%	100%
	1.0%	100%	100%	100%

## Data Availability

The data are contained within the article.
